# Decreased complexity of glucose dynamics preceding the onset of diabetes in mice and rats

**DOI:** 10.1371/journal.pone.0182810

**Published:** 2017-09-06

**Authors:** Xiaohua Douglas Zhang, David Pechter, Liming Yang, Xiaoli Ping, Zuliang Yao, Rumin Zhang, Xiaolan Shen, Nina Xiaoyan Li, Jonathan Connick, Andrea R. Nawrocki, Manu Chakravarthy, Cai Li

**Affiliations:** 1 Department of BARDS, Merck Research Laboratories, Kenilworth, New Jersey, United States of America; 2 Department of Pharmacology, Merck Research Laboratories, Kenilworth, New Jersey, United States of America; 3 Department of Diabetes, Merck Research Laboratories, Kenilworth, New Jersey, United States of America; 4 Department of Laboratories Animal Resources, Merck Research Laboratories, Kenilworth, New Jersey, United States of America; 5 Department of Translational Pharmacology, Merck Research Laboratories, Kenilworth, New Jersey, United States of America; Western University of Health Sciences, UNITED STATES

## Abstract

Continuous glucose monitoring (CGM) is a platform to measure blood glucose (BG) levels continuously in real time with high enough resolution to document their underlying fluctuations. Multiscale entropy (MSE) analysis has been proposed as a measure of time-series complexity, and when applied to clinical CGM data, MSE analysis revealed that diabetic patients have lower MSE complexity in their BG time series than healthy subjects. To determine if the clinical observations on complexity of glucose dynamics can be back-translated to relevant preclinical species used routinely in diabetes drug discovery, we performed CGM in both mouse (*ob/ob*) and rat (Zucker Diabetic Fatty, ZDF) models of diabetes. We demonstrate that similar to human data, the complexity of glucose dynamics is also decreased in diabetic mice and rats. We show that low complexity of glucose dynamics is not simply a reflection of high glucose values, but rather reflective of the underlying disease state (i.e. diabetes). Finally, we demonstrate for the first time that the complexity of glucose fluctuations in ZDF rats, as probed by MSE analysis, is decreased prior to the onset of overt diabetes, although complexity undergoes further decline during the transition to frank diabetes. Our study suggests that MSE could serve as a novel biomarker for the progression to diabetes and that complexity studies in preclinical models could offer a new paradigm for early differentiation, and thereby, selection of appropriate clinical candidate molecules to be tested in human clinical trials.

## Introduction

Currently, there are several methods to measure blood glucose levels under basal conditions and under states of physiologic and pharmacologic perturbations. However, many of these approaches have limitations: large blood volumes and frequent sampling generally only provide a snapshot of glycemic variation. Even when sampled frequently, these methodologies provide limited information on indices of glycemic variability and in particular, nocturnal glycemic events. Continuous glucose monitoring (CGM) is able to provide real time glucose data around the clock and over many days, while being minimally invasive and requiring negligible sampling volumes. Indeed the availability of CGM in the clinic has been hailed to as a revolutionary development in diabetes management [1]. As an example, two recent studies demonstrated that among patients with type 1 diabetes treated with multiple daily insulin injections, the use of CGM compared with conventional treatment resulted in lower HbA1c levels [2, 3]. Further, the use of CGM without confirmatory blood glucose monitoring measurements was as safe and effective as using CGM adjunctive to blood glucose monitoring in well-controlled adults with type 1 diabetes [4]. In aggregate, these recent clinical CGM data will likely catalyze the wider adoption of this technology to better manage glucose levels of diabetic patients [5].

As an example, monitoring glycemic variability continuously around the clock in diabetic patients taking different medications provided a much more complete picture on the glucose effects of these agents [6–8]. Monitoring changes in average glucose levels during a 24-hr period revealed that the DPP-4 inhibitor sitagliptin significantly lowered 24-h as well as daytime mean glucose levels before breakfast and lunch, compared with the α-glucosidase inhibitor voglibose, whereas the time from before dinner to peak postprandial glucose levels was significantly longer with voglibose compared with sitagliptin [7]. Data like these have the potential to help further understand the pharmacology of different classes of agents for a more optimal glucose control.

However, despite tens of thousands of data points being collected during CGM, the full spectrum of information encoded in such data is only just beginning to be elucidated. One of the tools that has been developed for characterizing physiological time series is multiscale entropy (MSE) and was succinctly summarized as follows[9], “MSE is based on the simple observation that complex physical and biologic systems generally exhibit dynamics that are far from the extrema of perfect regularity and complete randomness. Instead, complex dynamics typically reveal structure on multiple spatial and temporal scales. These multiscale features, ignored by conventional entropy calculations, are explicitly addressed in the MSE algorithm.” The MSE algorithm is freely available on-line as C-code, along with many related data resources[10]. MSE was developed to address limitations of earlier entropy measures and has been applied to develop a range of biological time series, such as cardiac inter-beat intervals and clinical CGM data[11, 12]. In the context of CGM, it was discovered that glucose levels in healthy or diabetic subjects are not constant but undergo small fluctuations constantly, and the information encoded in these fluctuations is significantly less complex in those with diabetes [12, 13]. Thus, while the actual levels of glucose are high in the diabetic state, complexity of glucose fluctuations is low. The complexity of glucose dynamics in CGM data is determined by MSE analysis, which considers the sample entropy, a statistical measure of irregularity or randomness, over multiple levels of granularity [11, 14]. Larger sample entropy values over multiple time scales indicate greater complexity. Detrended fluctuation analysis of 206 patients with essential hypertension, obesity or having a first-degree relative with a diagnosis of diabetes suggested a prognostic value of such analysis for predicting the development of type 2 diabetes [15, 16].

While CGM has been available in the clinic for more than a decade[1], it was not until 2014 that such a device became available for preclinical models [17]. Developed for preclinical applications, the HD-XG model to perform CGM employs an electrochemical glucose oxidase sensor placed directly in an artery. Glucose oxidase within the sensor serves as a catalyst to convert glucose and oxygen into gluconic acid and hydrogen peroxide, which in turn interacts with a noble metal electrode to give up electrons and create a current proportional to the amount of glucose available. The availability of chronic CGM options in preclinical species should allow detailed interrogation of CGM data to support diabetes drug discovery. This novel telemetry device from DSI is designed for continuous monitoring of temperature, locomotor activity, and plasma glucose levels in the arterial blood of rodents.

Given the potential of glucose complexity studies as a new preclinical and translational tool to drive innovation and develop differentiated diabetes therapies, it is critical to first determine whether there is any difference in the complexity of glucose dynamics between healthy and diabetic animals. Thus, in this study, we aimed to determine if reduced complexity of glucose dynamics seen in patients with diabetes also holds true in two widely used diabetes animal models, the C57BL/6 *ob/ob* mouse and the Zucker Diabetic Fatty (ZDF) rat.

## Materials and methods

### Animals

All testing protocols were reviewed and approved by the MRL Institutional Animal Care and Use Committees in Rahway and Kenilworth, NJ. The Guide for the Care and Use of Laboratory Animals was followed in the conduct of all animal studies. Animals were maintained on a 12 h/12 h light-dark cycle with free access to food and water in an environment with temperature maintained at 22°C. Three CGM experiments were performed either in *ob/ob* mice (Experiment 1) or ZDF rats (Experiments 2 and 3). At the end of the experiments, animal were euthanized with carbon dioxide and euthanasia confirmed, following harmonized IACUC guidelines adopted at Merck Research Laboratories.

Telemetry device provides direct continuous blood glucose readings along with temperature and activity for 4 weeks or longer. Each rat was surgically implanted with glucose sensors in the abdominal descending aorta and the telemetry device placed in the intraperitoneal cavity. In the mouse, glucose sensors were placed in the carotid artery and the device placed on the back subcutaneously. Continuous glucose readings were recorded with the Dataquest A.R.T.v.4.35 data acquisition system for at least 28 days with periodic calibrations. Glucose tolerance tests were performed for multi-point calibrations by administering dextrose to 2–5 g/kg, po or ip. Daily and GTT reference values were recorded with a non-clinical version of the StatStrip Xpress glucometer and strips (Nova Biomedical, Waltham, MA) capable of measuring blood glucose levels up to 900 mg/dL. Reference meter values were used for calibration in Microsoft Excel based on the initial GTT data with baseline corrections and then on the daily meter values thereafter.

### Experiment 1

Six male lean C57BL/6 mice and six age- and sex-matched *ob/ob* mice were ordered from the Jackson Laboratory (Bar Harbor, ME); date of birth of the mice were approximately on Dec. 29, 2015. Mice were received on Feb. 17, 2016 and acclimated. Surgery to implant the sensors was on Mar. 7, 2016. Data acquisition started on Mar. 8, 2016 at the age of 72 days. Glucose was recorded from age 72 days to age 84 days.

### Experiment 2 and 3

Four male ZDF and four age- and sex-matched lean control rats were ordered from CRL (Williamston, MA) at the age of 11 weeks (Experiment 2) and 5 weeks (Experiment 3), respectively. Younger rats at 5 weeks of age were used to determine the timing of the decrease of MSE in ZDF rats. The earliest age ZDF rats become commercially available is at 5 weeks, when their glucose levels are still indistinguishable from lean controls. Surgery to implant the sensors for Experiment 2 was conducted on Oct. 28–29, 2015 (DOB of rats: week of Aug. 5, 2015) and CGM device turned on on Nov. 5, 2015 at 0:00 am (midnight is 12:00 AM; noon is 12:00 PM). Single point calibrations were performed on Nov. 5, 12, 19, and 23, as well as Dec. 4 and 7. For Experiment 3, data acquisition started on Mar. 3, 2016 at the age of 38 days.

### Calibration of the HD-XG glucose sensor

An initial multi-point calibration was performed within 7 days of surgery and required taking at least two reference points at glucose levels that vary by at least 100 mg/dL. This was accomplished by an oral glucose tolerance test in the lean mice (5 g/kg of 50% dextrose given in a volume of 10 mL/kg) or an insulin tolerance test on *ob/ob* mice (1 U/mL of insulin dosed at 5 mL/kg to a final dose of 5 U/kg). Periodic reference measurements of tail blood glucose were performed following the initial multi-point calibration and were taken at least twice per week throughout the course of the study. BW was also taken when glucose levels were measured. Animals were monitored continuously for at least 28 days. For ZDF and lean control rats, reference measurements were taken at a frequency of approximately weekly on Nov. 5, 12, 19, 23, and Dec. 4 and 7, following surgery on Oct. 28–29. An ipGTT multi-point calibration was performed on Dec. 4, 2015 by IP delivery of 2 g/kg of glucose and glucose levels read at 10, 20, 30 minutes and at 1 hour after glucose was delivered.

### Data analysis

For calculating MSE we essentially adopted the C codes from Costa et al [12] although we made a few minor changes for input and output to make it easier for them to be called in R. We wrapped the execution of these C codes into our R functions and then conducted analysis in R. Missing values commonly exist in the CGM data because animals need to be removed from the cage for glucose sensor calibrations or cage changes. The C codes from Costa et al are not able to handle missing values. Because there were only a few intervals of missing values for each animal and each interval was only a few observations long, we simply omitted those intervals from the data series before applying the calculation in the C codes. For all analyses, we used MSE parameters of m = 2 and r = 0.15 (http://www.physionet.org/)[12].

The CGM device record one value every 10 seconds in all the three experiments. For Experiment 1, MSE analysis of CGM fluctuation was performed on data collected during the two-week period from age 72 days to age 84 days. For Experiment 2, MSE analysis was performed on data collected from Day 3 to Day 14, counting the start of CGM as Day 1. For Experiment 3, MSE analysis was performed on the glucose data separately for the period from age 38 days to 41 days, and the period from 42 days to 48 days. This was based on the results that the daily average glucose level in the young ZDF rat is not significantly different from the young lean rat in each day before the age of 42 days but is significantly different in each day starting the age of 42 days.

## Results

### Multiscale entropy is decreased in diabetic *ob/ob* mice compared to non-diabetic lean controls

**Fig 1:**
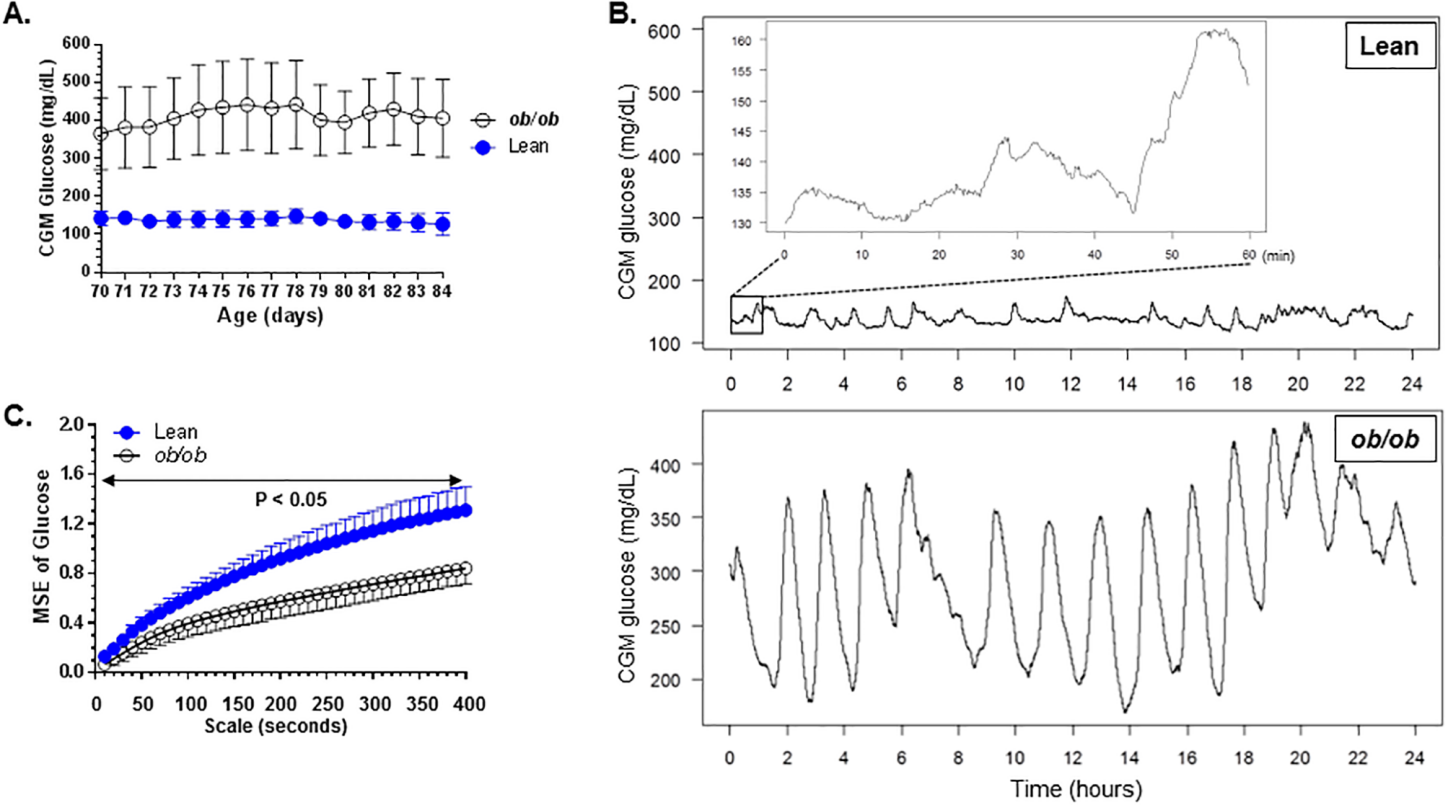
(**A**). Glucose levels of *ob/ob* mice and age-matched lean controls used for the study. Error bars, standard deviation. N = 6 mice/grouop. (**B**). Time series of blood glucose levels derived from CGM recordings of a non-diabetic lean mouse and a diabetic *ob/ob* mouse during a 24-hr period (age day 70 to 71). The inset is magnification of a one hour glucose data in a non-diabetic lean mouse. (**C**). Multiscale entropy analysis on converted glucose levels over Day 72 to Day 84 in *ob/ob* mice and lean controls (mean and standard deviation). The P values in Fig 1C were calculated by performing unpaired t-tests for differences in mean sample entropy between the two groups of mice at the time scales indicated.

At the age of 72 days, glucose levels of *ob/ob* mice and control littermates were 365.1 +/- 94.6 mg/dL (Mean +/- SD, n = 6) and 141.9 +/- 18.7 mg/dL (n = 6), respectively (Fig 1A). There are significant differences in glucose levels between the two groups for each day of the Day 72 to Day 84 period. CGM data were recorded at a frequency of one data point for every ten seconds. Data from Day 72 to Day 84 were used for MSE analysis (from 2016/03/10 16:00:00 to 2016/03/23 17:05:50). To allow visual assessment of the complexity of the fine structure of the glucose variability, the fluctuations in glucose values during a 24-hour period as well as the magnification of a one hour period is shown (Fig 1B and inset) for a healthy mouse. The time scales of the fluctuations depicted in the insert are closer to those considered for MSE analysis. The MSE results for converted glucose levels for scales 10 to 400 seconds for the lean and *ob/ob* mice over Day 72 to Day 84 are shown Fig 1C. The entropy of CGM time series was significantly (p<0.05, t-test) lower in the group of *ob/ob* mice than in healthy controls (Fig 1C) for time scales ranging from 10 to 400 seconds. These results indicate that dynamical complexity of CGM fluctuations was higher in lean controls than in *ob/ob* mice.

### Multiscale entropy is decreased in diabetic ZDF rats compared to non-diabetic lean controls

**Fig 2:**
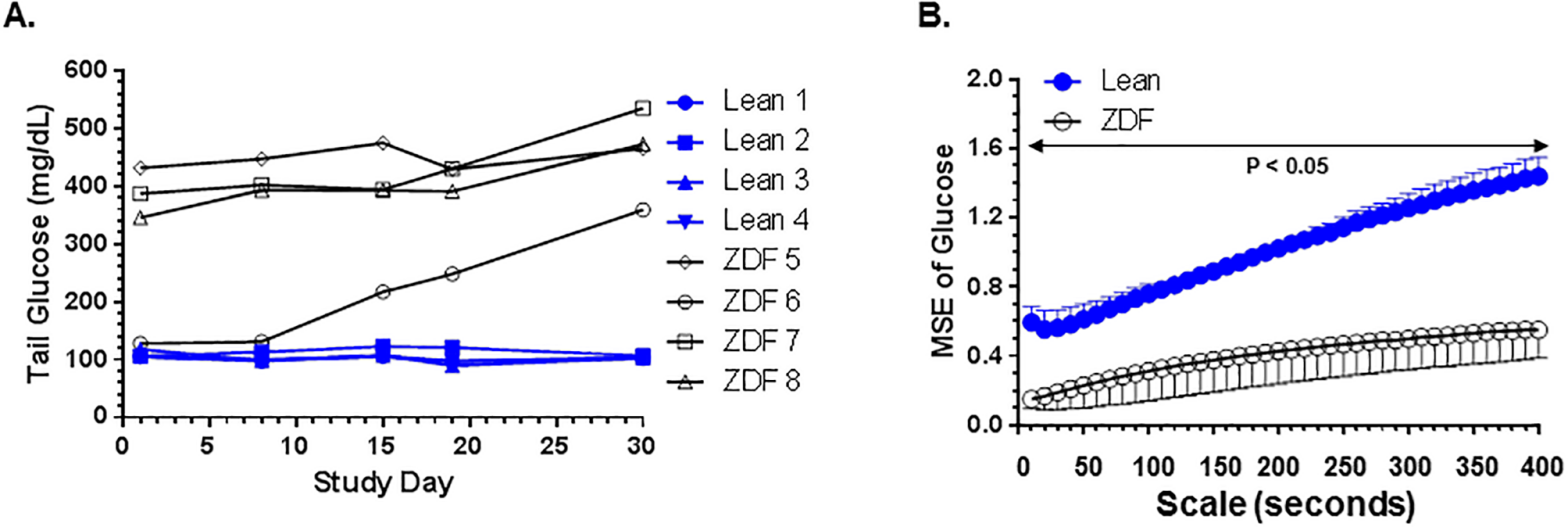
(**A**). Glucose levels of diabetic ZDF rats and age-matched lean controls used for the study. Glucose levels of each rat is shown from Day 1 to Day 30 of study. N = 4 rats/grouop. (**B**). Multiscale entropy analysis on converted glucose levels over Day 3 to Day 14 in ZDF rats and lean littermate controls (mean and standard deviation). The P values in Fig 2B were calculated by performing unpaired t-tests for differences in mean sample entropy between the two groups of mice at the times scales indicated.

Similar to the *ob/ob* mouse study describe above, multiscale entropy was determined in diabetic ZDF rats and lean littermate controls. At the start of the experiment on Day 1, glucose levels of ZDF rats and control lean littermates were 323.0 +/- 134.7 mg/dL (Mean +/- SD, n = 4) and 108.4 +/- 6.1 mg/dL (n = 4), respectively (Fig 2A). Glucose values from Day 3 to Day 14 were used for MSE analysis. The entropy of CGM time series was significantly (p<0.05, t-test) lower in diabetic ZDF rats than in lean littermate controls (Fig 2B). These results indicate that dynamical complexity of CGM fluctuations was higher in lean controls than in diabetic ZDF rats (Fig 2B). The p-values of unpaired t test for testing no difference between the lean and ZDF rats at scale 10, 20, 30, 40 are 0.01, 0.006, 0.0013, 0.0003, respectively, all being very small. The MSE in the lean rats is significantly higher than that in the ZDF rats, demonstrating that the complexity of glucose dynamics in a lean rat is significantly higher than that in a ZDF rat.

### Multiscale entropy is decreased prior to the onset of diabetes and undergoes further rapid decline during the onset of frank diabetes in ZDF rats

We noted that glucose levels of one of the four ZDF rats were only starting to increase during the period of MSE analysis (Fig 2A, rat ZDF 5). However, entropy values of ZDF 5 were not different from the other three ZDF rats that were already frankly diabetic during the same period (Fig 2B). This observation raised the possibility that decrease in complexity of glucose dynamics in a genetic model of diabetes could occur prior to the onset of overt diabetes.

**Fig 3:**
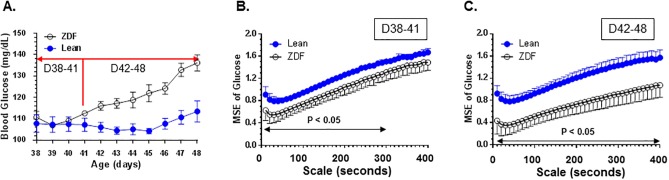
(**A**). Glucose levels of young ZDF rats and age-matched lean controls used for the study. Daily mean glucose levels of ZDF rats or controls from the age of 38 days through the age of 48 days were plotted. N = 4 rats/grouop. D38-41, age Day 38 to Day 41; D42-48, age Day 42 to Day 48. Glucose levels were not significantly different between ZDF rats and lean controls during the age of Day 38 to Day 41 (D38-41) but were statistically different during the age of Day 42 to Day 48 (D42-48). (**B**). Reduced multiscale entropy in ZDF rats compared with age-matched controls when glucose levels were not different at the age of D38-41. (**C**). Further decline of multiscale entropy in ZDF rats compared with age-matched controls during the transition to hyperglycemia at the age of D42-48. The P values in Fig 3B and 3C were calculated by performing unpaired t-tests for differences in mean sample entropy between the two groups of mice at the times scales indicated.

These young rats were implanted with CGM sensors and glucose levels recorded at the age of Day 38. From Day 38 to Day 41, glucose levels were not statistically different. From Day 42 to Day 48, glucose levels of ZDF rats started to rise and become statistically different from lean controls (Fig 3A), although the values of glucose are still much lower than that seen in frankly diabetic ZDF rats (Fig 2A).

MSE values were calculated during the period of Day 38–41, when glucose levels of ZDF rats were not different from lean rats and during the period of Day 42–48, when the glucose levels of ZDF rats just started to become significantly different from lean controls. Though glucose levels have not separated between ZDF rats and lean controls between D38 and D41 (Fig 3A), complexity of glucose dynamics is already significantly different (Fig 3B). Further, when ZDF rats progress to become hyperglycemic, complexity of glucose dynamics declines further with the onset of overt diabetes (Fig 3C).

## Discussion

In this report, we demonstrated that complexity of glucose dynamics, as determined by MSE analysis, is reduced in diabetes in two commonly used preclinical models, the *ob/ob* mouse as well as the ZDF rat. This observation noted in 2 distinct species was similar to that found in humans [12, 13], suggesting that MSE is likely an evolutionarily conserved feature. A novel finding of this study was the decrease of MSE (i.e. decreased complexity of glucose dynamics) *prior* to the onset of overt diabetes, which to the best of our knowledge has not been reported to date in any preclinical species. This observation suggests a predictive value of MSE analysis as a potential new biomarker of diabetes development. The rapid decline of MSE while transitioning to diabetes suggests that the metabolic changes during this period may have a direct impact on MSE. This discovery supports a new paradigm [12] for the diagnosis and/or treatment of diabetes by controlling the complexity of glucose dynamics. For example, treatments could be targeted to restore the complexity of glucose dynamics in pre-diabetes to prevent the onset of overt diabetes. It could also lead to better diagnostics using complexity or by combining complexity with current static measures (e.g. FPG or HbA1c), instead of just these static measures alone, the current standard-of-care.

Our current finding on loss of complexity of glucose dynamics in diabetes is consistent with earlier studies in other physiological systems. There is a large body of literature demonstrating metabolic inflexibility when one transitions from healthy to insulin resistance or frank diabetes in various tissues [18–21]. These data are similar in nature to the MSE analysis reported here. The transition from healthy to diabetes is likely the manifestation of loss of metabolic flexibility, with MSE analysis being a method to quantify the differences in healthy and diabetic states.

Compared with the clinical CGM, the DSI HD-XG glucose sensor may offer a number of advantages. Specifically, clinical CGM devices require calibrations every 12 hours, and the sensors typically last 3–7 days only. In contrast, the DSI HD-XG sensor required calibrations no more than twice a week and lasts for up to two months, allowing the collection of uninterrupted data during this entire period. Most importantly, HD-XG reports blood glucose data every ten seconds from arterial blood, while clinical CGM generates data at an interval every five minutes from interstitial fluid and not from blood vessel. Glucose in the interstitial fluid may not accurately reflect blood glucose levels in real time, especially during periods of acute glucose changes such as after a meal or during an acute stress response. These advantages of the preclinical HD-XG system allowed the comparison of sample multiscale entropies from a very small number of animals.

In an observational study of 37 non-diabetic volunteers age 12–65 years and 49 adults with longstanding type 1 diabetes, multivariate analysis of CGM data revealed low complexity of CGM profiles associated with insulin resistance in both non-diabetic subjects and patients with type 1 diabetes [22]. In non-diabetic subjects, low complexity could be an earlier marker of glucose regulation failure [15, 16, 22].

Physiological data captured in a time series is often fractal in nature. The complexities of such data are amenable for MSE analysis, which has led to a much deeper understanding of these complex physiological processes [11], ranging from heart beats, intracranial pressure, as well as blood glucose control. In one instance, the complexity of intracranial pressure correlates with outcome after traumatic brain injury [23]. Another study demonstrated that heart rate complexity is reduced with a significant decreasing trend as assessed by R-R interval entropy prior to the onset of atrial fibrillation [24]. Along with the findings of lower complexity of glucose in diabetes, additional physiological readouts expressed as time series may add to our collection of existing associations between complexity and health [25].

The observed decreases of complexity in both diabetic rodents (*ob/ob* mice and ZDF rats) as well as diabetic patients suggest that multiscale entropy analysis could serve as a novel biomarker for diabetes development, as well as in the selection of potential therapies earlier in the drug development process. Decrease in complexity prior to the onset of diabetes in pre-diabetic ZDF rats suggests that multiscale entropy values could serve as a predictive biomarker of diabetes development. An interesting and yet unanswered question is the directional changes in multiscale entropy upon pharmacological treatment to normalize glucose levels. For example, will complexity be restored upon glucose lowering by different classes of diabetes therapies? If complexity does increase with glucose lowering, will the increase occur prior to, in parallel with, or subsequent to an observable decrease of glucose levels? The answers to these fascinating questions can substantively impact diabetes drug discovery. For instance, by incorporating MSE analyses in routine preclinical evaluation, it is conceivable to select one drug vs. another by choosing the one that restores complexity for clinical development, even if both drugs were to lower BG to similar levels as measured by conventional methods such as FPG or HbA1c. Differentiation between drug candidates could also be considered based on the magnitude of complexity restoration. Our work in these preclinical species is in full agreement with the earlier work of Costa et al that suggested “…these findings support consideration of a new framework, dynamical glucometry, to guide mechanistic research and to help assess and compare therapeutic interventions, which should enhance complexity of glucose fluctuations and not just lower mean and variance of blood glucose levels"[12].

In summary, while the need and value of a novel diabetes therapy to restore complexity of glucose dynamics on top of standard BG lowering remains to be established, our study and prior work suggest that the complexity of glucose dynamics may have the potential to become a new preclinical and translational tool to drive innovation to develop much-needed differentiated therapeutics for the prevention and treatment of diabetes.
